# Clinical characteristics and short-term outcomes of multisystem inflammatory syndrome in a country with a high prevalence of KD

**DOI:** 10.3389/fped.2023.1088529

**Published:** 2023-02-14

**Authors:** Sung Doo You, Jin Ho Kim, Jihye You

**Affiliations:** ^1^ Department of Pediatrics, Jeonbuk National University Children's Hospital, Jeonju, Korea; ^2^ Research Institute of Clinical Medicine of Jeonbuk National University, Jeonju, Korea

**Keywords:** Pediatric multisystem inflammatory disease, COVID-19 related, COVID-19, echocardiogaphy, kawasaki disease, SARS-CoV-2, albumin

## Abstract

**Background:**

The COVID-19 pandemic has spread continuously. Multisystem inflammatory syndrome in children (MIS-C), like Kawasaki disease (KD), is a potentially severe illness in children that appears to be a delayed, post-infectious complication of COVID-19. However, based on the relatively low MIS-C prevalence and high KD prevalence in Asian children, the clinical features of MIS-C are not fully recognized, especially after the spread of the Omicron variant. Here, we aimed to identify the clinical characteristics of MIS-C in a country with high KD prevalence.

**Methods:**

We retrospectively analyzed 98 children diagnosed with KD and MIS-C admitted to Jeonbuk National University Hospital between January 1, 2021, and October 15, 2022. Twenty-two patients were diagnosed with MIS-C, following CDC diagnostic criteria for MIS-C. We reviewed medical records for clinical features, laboratory findings, and echocardiography.

**Results:**

Age, height, and weight were higher for patients with MIS-C than for those with KD. Lymphocytes percentage was lower, and the segmented neutrophil percentage was higher in the MIS-C group. The inflammation marker C-reactive protein was higher in the MIS-C group. Prothrombin time was prolonged in the MIS-C group. Albumin level was lower in the MIS-C group. The MIS-C group had lower potassium, phosphorus, chloride, and total calcium. Twenty-five percent of patients diagnosed with MIS-C had positive RT-PCR, and all the patients were N-type SARS-CoV-2 antibody-positive. Albumin ≤3.85 g/dl effectively predicted MIS-C. Regarding echocardiography, the right coronary artery *z*-score, the absolute value of apical 4-chamber left ventricle longitudinal strain, and the ejection fraction (EF) was significantly lower in the MIS-C group. A month after diagnosis using echocardiographic data, all coronary artery *z*-scores had reduced significantly. EF and fractional shortening (FS) also improved one month after diagnosis.

**Conclusion:**

Albumin values can differentiate MIS-C and KD. In addition, a decrease in the absolute LV longitudinal strain value, EF, and FS was observed in the MIS-C group using echocardiography. Coronary artery dilatation was not evident at the initial diagnosis; however, a change in coronary artery size, EF, and FS was observed on follow-up echocardiography a month after diagnosis.

## Introduction

The coronavirus infection disease 2019 (COVID-19) is caused by severe acute respiratory syndrome coronavirus 2 (SARS-CoV-2). This novel coronavirus rapidly reached pandemic proportions. Overall, COVID-19 is mild in pediatric patients (1). However, reports from the UK documented its presentation as incomplete Kawasaki disease (KD) or toxic shock syndrome, and similarly affected children have been reported in other parts of the world (2). This condition was termed multisystem inflammatory syndrome in children (MIS-C). MIS-C symptoms include persistent fever, gastrointestinal disturbances (abdominal pain, vomiting, and diarrhea), mucocutaneous symptoms (such as conjunctivitis and mucosal changes), and dermatologic symptoms (such as rash and edema to the extremities) (3, 4). MIS-C occurs in <1% of children with COVID-19 history and is common in white and black populations but relatively uncommon in Asian patients (4). Post-infectious immune dysregulations might be the pathophysiology of MIS-C. Preliminary studies suggest that patients with MIS-C have persistent immunoglobulin G (IgG) antibodies with high inflammatory monocyte-activating ability, T cell lymphopenia, and CD8+ T cell activation (4, 5).

KD is an acute onset systemic vasculitis, which involves small-to-medium-sized arteries. This febrile disease is the most common heart disease in pediatric patients (1). The clinical manifestations are skin rash, bilateral conjunctivitis, cervical lymphadenopathy, oral mucosal changes, and swelling or redness of the extremities (6). Coronary artery aneurysm or dilatation, aortic root dilatation, and myocarditis may occur in the cardiac manifestation (1). KD prevalence is highest in East Asian countries despite being a worldwide illness (7). Approximately 80% of KD occurs in pediatric patients of <5 years (8). KD pathogenesis has not been fully identified; however, infections with environmental and genetic factors might be triggers for the disease (9–11).

Due to clinical manifestation similarity, distinguishing MIS-C from KD is challenging, although there is a specific immunophenotype in MIS-C (4, 5). In general, MIS-C occurs more commonly in older patients than KD, and gastrointestinal symptoms, shock, and lymphopenia are more common in MIS-C (4, 5). However, MIS-C is relatively rare in Asia, and KD occurs most frequently in east Asia, making MIS-C hard to distinguish from KD in grey-zone kids. Besides, COVID-19 history is common in pediatric patients because SARS-CoV-2 rapidly spreads in Asian countries; therefore, the differentiation points become insignificant and accurate diagnosis becomes even more challenging. In areas with high KD incidence, the most clinically helpful factors to differentiate MIS-C from KD is unknown. Furthermore, the clinical manifestations of MIS-C in a country with high KD prevalence are not properly identified. Herein, the authors aimed to determine MIS-C manifestations in a country with the second highest KD incidence, compare the clinical features of MIS-C with KD, and find the most effective distinguishing factors in a clinical setting, especially in high KD prevalence where grey-zone patients exist.

## Methods

### Study design

**Figure 1:**
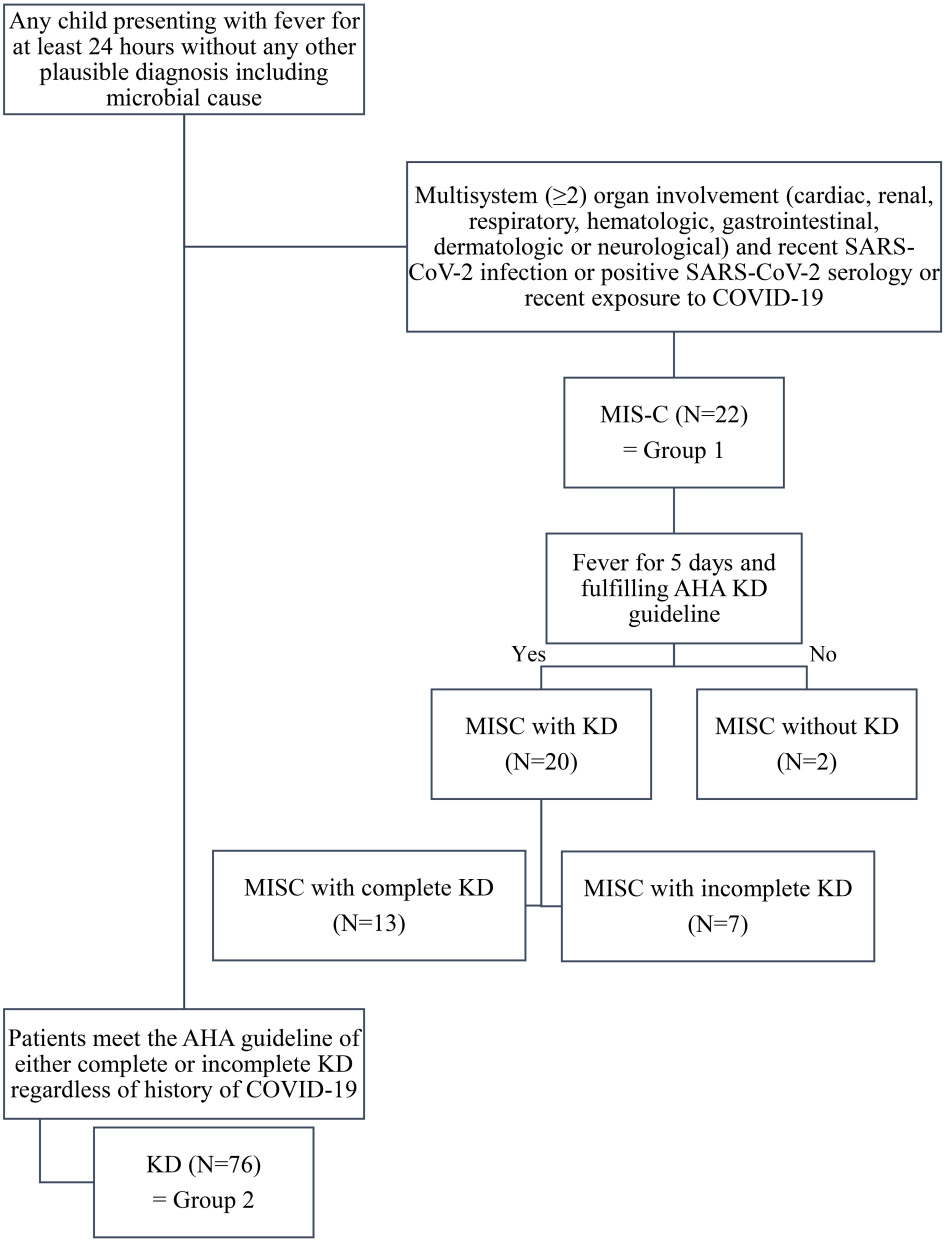
Flow chart of inclusion of patients and grouping.

All patients under 18 years admitted to Jeonbuk National University Children's Hospital, a tertiary referral center in the Republic of Korea for KD or MIS-C, between January 1, 2021, and October 15, 2022, were enrolled. KD was diagnosed using the American Heart Association (AHA) criteria (6). Clinical symptoms and laboratory investigations were used for diagnosis. MIS-C was diagnosed using the US Center for Disease Control (CDC) criteria (12). In total, 98 patients were enrolled in this study. Twenty-two patients were diagnosed with MIS-C, constituting “group 1” in this study. Among these patients, 20 (90.9%) fulfilled the AHA KD criteria. Complete KD was diagnosed in 13 patients (59.1%) with MIS-C. The remaining 76 patients did not meet the diagnostic criteria for MIS-C and were assigned to “group 2.” The flow chart showing the enrolled is given in Figure 1. We reviewed the patient's demographic data, clinical signs of KD, cardiac, renal, respiratory, mucocutaneous, dermatologic, hematologic, gastrointestinal, and neurologic symptoms of MIS-C, COVID-19 history, laboratory data, echocardiographic findings, responsiveness to intravenous immunoglobulin (IVIG), clinical course, and echocardiographic results a month after diagnosis. BMI percentile was calculated based on the CDC growth chart (13).

A refractory case of MISC or KD was defined as a case that needed secondary treatment. On the contrary, treatment failure on the first IVIG was defined by persistent fever for 36 h after IVIG only in patients primarily treated with IVIG.

### Serologic test of SARS-CoV-2 antibody

Serologic tests of SARS-CoV-2 nucleocapsid (N) and spike (S) proteins were performed, and IgG and IgM antibodies were analyzed. The blood was analyzed using a chemiluminescence immunoassay (CLIA), and a 1.0 cut-off index was used, equivalent to 0.80 U/ml, using electrochemiluminescence immunoassay (ECLIA). SARS-CoV-2 serologic test was conducted in 66 patients (67.2%). S antibody was positive in 37 patients (56.1%), and N antibody was positive in 36 (54.5%). Twenty-seven (73.0%) patients with positive N antibodies had COVID-19 history. The mean interval between COVID-19 to the first manifestation of MIS-C or KD was 86.0 (12–240) days. In 5 (13.9%) patients with positive N antibodies, SARS-CoV-2 PCR was positive.

### Echocardiography

The Philips IE33 system (Phillips Medical Systems, Andover, MA, USA) was used for echocardiography. A pediatric cardiologist (J.Y.) performed the echocardiography, and three-cycle loop heartbeats were recorded. Transducers were chosen based on the patient's physical development. Due to race similarity, the *z*-scores of coronary arteries were calculated based on the study of Kobayashi et al. (14). The diastolic left ventricular internal diameter (LVIDd) *z*-scores were calculated using the method published by Patterson et al. (15).

All echocardiographic data were interpreted using aCMQ. The myocardium was manually traced at the end-diastolic point on a single frame, and the machine estimated the 2-dimensional (2D) longitudinal strain. We only analyzed the longitudinal strain of apical 4-chamber images. Since a single experienced cardiologist conducted the echocardiography and calculated the strain, only an interobserver reproducibility test was performed.

### Statistical analysis

SPSS Statistics for Windows, version 27.0 (IBM Corp., Armonk, NY, USA) was used for statistical analysis. *P*-value < 0.05 was considered significant. Numerical data are stated as mean ± standard deviation and categorical data as frequency (%). All the patients were divided into 2 groups according to MIS-C diagnosis. Patients in group 1 were diagnosed with MIS-C irrespective of KD diagnosis, and those in group 2 were diagnosed with only KD. Between both groups, unpaired *t*-tests, Fisher's exact test, and Pearson *χ*^2^ tests compared the data. A ROC curve was drawn using the results of compared data to investigate the MIS-C predictors. Univariate and multivariate logistic regression analyses determined the effectiveness of the seven predictors with the highest area under the curve (AUC) as the categorical variable (1 for MIS-C and 0 for only KD) in predicting MIS-C. R version 4.2.2. (R Foundation for Statistical Computing, Vienna, Austria) was used for propensity score–matched analysis. Finally, the paired *t*-test was used to compare echocardiography between the two time points (at diagnosis vs. 1 month after diagnosis).

## Results

Demographic and clinical characteristics and laboratory findings of each group are presented in Table 1. The mean age (8.6 ± 3.8 vs. 2.3 ± 2.8 years, *P *< 0.001), height (134.9 ± 25.2 vs. 87.5 ± 21.4 cm, *P *< 0.001), and weight (35.0 ± 17.6 vs. 13.7 ± 8.6 kg, *P *< 0.001) were significantly higher in group 1 than in group 2. There was no difference in sex and BMI percentile between the two groups. In clinical characteristics, rash and extremity changes were observed less frequently in group 1 (*P *< 0.001 and *P *= 0.013, respectively). Regarding complete blood cell analysis, mean corpuscular volume (MCV) of red blood cell (RBC) (84.5 ± 4.7 vs. 81.4 ± 3.9 fl, *P *= 0.003), segmented neutrophils percentage (76.6 ± 9.5 vs. 52.2 ± 20.5%, *P *< 0.001) were predominant in group 1, whereas lymphocytes percentage (16.0 ± 8.2 vs. 36.2 ± 18.4%, *P *< 0.001) was prominently lower in group 1 than that in group 2. No significant difference was observed in cardiac makers of both groups. Regarding inflammatory markers, C-reactive protein (CRP) levels were higher in group 1 (93.8 ± 54.8 vs. 46.4 ± 44.5 mg/L, *P *< 0.001). Additionally, prothrombin time (PT) was longer in group 1 than in group 2 (13.5 ± 1.6 vs. 11.9 ± 1.4 s, *P *= 0.006). In blood chemistry laboratory findings, albumin was lower in group 1 than in group 2 (3.6 ± 0.5 vs. 4.2 ± 0.5 g/dl, *P *< 0.001). In electrolyte analysis, potassium (4.0 ± 0.5 vs. 4.5 ± 0.6 mmol/L, *P *< 0.001), chloride (101.9 ± 3.8 vs. 104.2 ± 3.8 mmol/L, *P *= 0.011), phosphorus (3.7 ± 0.8 vs. 4.4 ± 0.9 mg/dl, *P *= 0.002), and calcium (8.8 ± 0.5 vs. 9.7 ± 0.6 mg/dl, *P *< 0.001) were lower in group 1 than in group 2. The positive rate of SARS-CoV-2 antibodies (100 vs. 41.3%, *P *< 0.001) and reverse transcription polymerase chain reaction (RT-PCR) (25.0 vs. 2.7%, *P *= 0.008) were higher in group 1 than in group 2.

**Table 1:** Demographic and clinical characteristics and laboratory findings of MIS-C and KD.

	Group 1 (*n* = 22)	Group 2 (*n* = 76)	*P*-value
Age (years)	8.6 ± 3.8	2.2 ± 2.8	**<0**.**001**
Male (%)	45.5	55.3	0.417
Height (cm)	134.9 ± 25.2	87.5 ± 21.4	**<0**.**001**
Weight (kg)	35.0 ± 17.6	13.7 ± 8.6	**<0**.**001**
BMI percentile	53.3 ± 36.5	60.0 ± 28.9	0.458
Duration of fever (days)	5.5 ± 2.3	5.5 ± 1.5	0.864
Rash (%)	63.6	96.1	**<0**.**001**
Conjunctivitis (%)	68.2	84.2	0.125
Oral mucosa or lips changes (%)	86.4	82.9	1.000
Extremity changes (%)	45.5	73.7	**0**.**013**
LN enlargement (%)	81.8	64.5	0.123
**Complete blood counts**			
WBC (×10^3^/ul) (4.0–10.0)	10.0 ± 5.3	11.0 ± 4.7	0.389
Hb (mg/dl) (12.0–16.0)	11.7 ± 1.1	11.3 ± 1.3	0.234
Hct (%) (37–48%)	35.6 ± 3.1	34.4 ± 3.7	0.169
MCV (fl) (81–99)	84.5 ± 4.7	81.4 ± 3.9	**0**.**003**
Plt (×10^3^/ul) (130–450)	251.1 ± 165.0	317.9 ± 151.5	0.077
Seg neutrophil (%) (50–75)	76.6 ± 9.5	52.2 ± 20.5	**<0**.**001**
Lymphocyte (%) (20–44)	16.0 ± 8.2	36.2 ± 18.4	**<0**.**001**
**Cardiac markers**			
Troponin-T (ng/ml) (0–0.1)	0.014 ± 0.005	0.021 ± 0.052	0.575
CK-MB (ng/ml) (0–2.88)	2.9 ± 4.1	2.2 ± 1.3	0.522
Pro-BNP (pg/ml) (0–113)	1276.9 ± 1426.9	1123.4 ± 1730.7	0.705
**Inflammatory markers**			
C-reactive protein (CRP) (mg/l) (0–5.0)	93.8 ± 54.8	46.4 ± 44.5	**<0**.**001**
ESR (mm/hr) (0–20)	36.1 ± 28.6	43.6 ± 24.7	0.235
Ferritin (ng/ml) (30–400)	361.7 ± 237.6	324.6 ± 497.8	0.865
**Coagulation factors**			
PT (s) (9.2–12.6)	13.5 ± 1.6	11.9 ± 1.4	**0**.**006**
aPTT (s) (24.8–36.1)	31.8 ± 4.3	33.0 ± 4.4	0.453
Fibrinogen (md/dl) (191–471)	460.0 ± 110.4	407.2 ± 173.9	0.386
D-dimer (mg/L) (0–0.49)	2.3 ± 1.9	1.9 ± 1.5	0.476
**Chemistry**			
AST (IU/L) (0–40)	56.9 ± 51.5	73.3 ± 121.5	0.540
ALT (IU/L) (0–41)	69.6 ± 112.5	63.8 ± 117.0	0.838
Total bilirubin (mg/dl) (0–1.2)	0.7 ± 0.7	0.4 ± 0.4	0.106
Albumin (g/dl) (3.8–5.4)	3.6 ± 0.5	4.2 ± 0.5	**<0**.**001**
BUN (mg/dl) (6–20)	9.9 ± 4.1	8.5 ± 3.8	0.125
**Electrolyte**			
Sodium (mmol/L) (136–145)	136.5 ± 2.8	137.2 ± 3.0	0.194
Potassium (mmol/L) (3.5–5.1)	4.0 ± 0.5	4.5 ± 0.6	**<0**.**001**
Chloride (mmol/L) (98–107)	101.9 ± 3.8	104.2 ± 3.8	**0**.**011**
Phosphorus (mg/dl) (3.1–6.0)	3.7 ± 0.8	4.4 ± 0.9	**0**.**002**
Total calcium (mg/dl) (8.8–10.8)	8.8 ± 0.5	9.7 ± 0.6	**<0**.**001**
**Molecular diagnostics**			
RT-PCR (%)	25.0	2.7	**0**.**008**
SARS-CoV-2 Ab (%)	100	41.3	**<0**.**001**

WBC, white blood cell; Hb, hemoglobin; Hct, hematocrit; MCV, mean corpuscular volume; Plt, *p*latelet; seg neutrophil, segmented neutrophil; ESR, erythrocyte sedimentation rate; PT, *p*rothrombin time; aPTT, activated partial thromboplastin time; AST, aspartate aminotransferase; ALT, alanine aminotransferase; BUN, blood urea nitrogen; CK, creatine kinase; LD, lactate dehydrogenase; RT-PCR, real-time reverse transcription polymerase chain reaction.

### Comparing the echocardiography data of both groups

The echocardiography findings of each group are presented in Table 2. Coronary arteries *z*-scores did not significantly differ between both groups except RCA *z*-score (0.8 ± 0.9 vs. 1.4 ± 1.4, *P *= 0.044). However, the absolute value of the apical 4-chamber longitudinal LV strain of group 1 was significantly lower than that of group 2 (−15.4 ± 3.3 vs. −17.8 ± 4.2%, *P *= 0.017). Also, LV EF by M-mode (65.1 ± 8.2 vs. 71.4 ± 6.2%, *P *< 0.001) and FS (35.6 ± 6.1 vs. 39.7 ± 5.1%, *P *= 0.002) of group 1 were lower than that of group 2. No patients had EF <55% in any of the groups.

**Table 2:** Results of echocardiographic examination.

	Group 1 (*n* = 22)	Group 2 (*n* = 76)	*P*-value
LMCA *z*-score	1.8 ± 0.9	2.0 ± 1.1	0.648
LAD *z*-score	1.3 ± 0.9	1.7 ± 1.1	0.076
LCX *z*-score	0.8 ± 1.0	0.7 ± 1.2	0.822
RCA *z*-score	0.8 ± 0.9	1.4 ± 1.4	**0** **.** **044**
LV strain (A4C) (%)	−15.4 ± 3.3	−17.8 ± 4.2	**0**.**017**
EF (%) (55–85)	65.1 ± 8.2	71.6 ± 6.2	**<0**.**001**
FS (%) (25–45)	35.6 ± 6.1	39.7 ± 5.1	**0**.**002**
LVIDd *z*-score	0.1 ± 0.8	0.2 ± 1.1	0.600

LMCA, left main coronary artery; LAD, left anterior descending artery; LCx, left circumflex artery; RCA, right coronary artery; LV, left ventricle; A4C, apical 4 chamber; FS, fractional shortening; LVIDd, diastolic left ventricular internal diameter.

### Prediction of MIS-C

**Figure 2:**
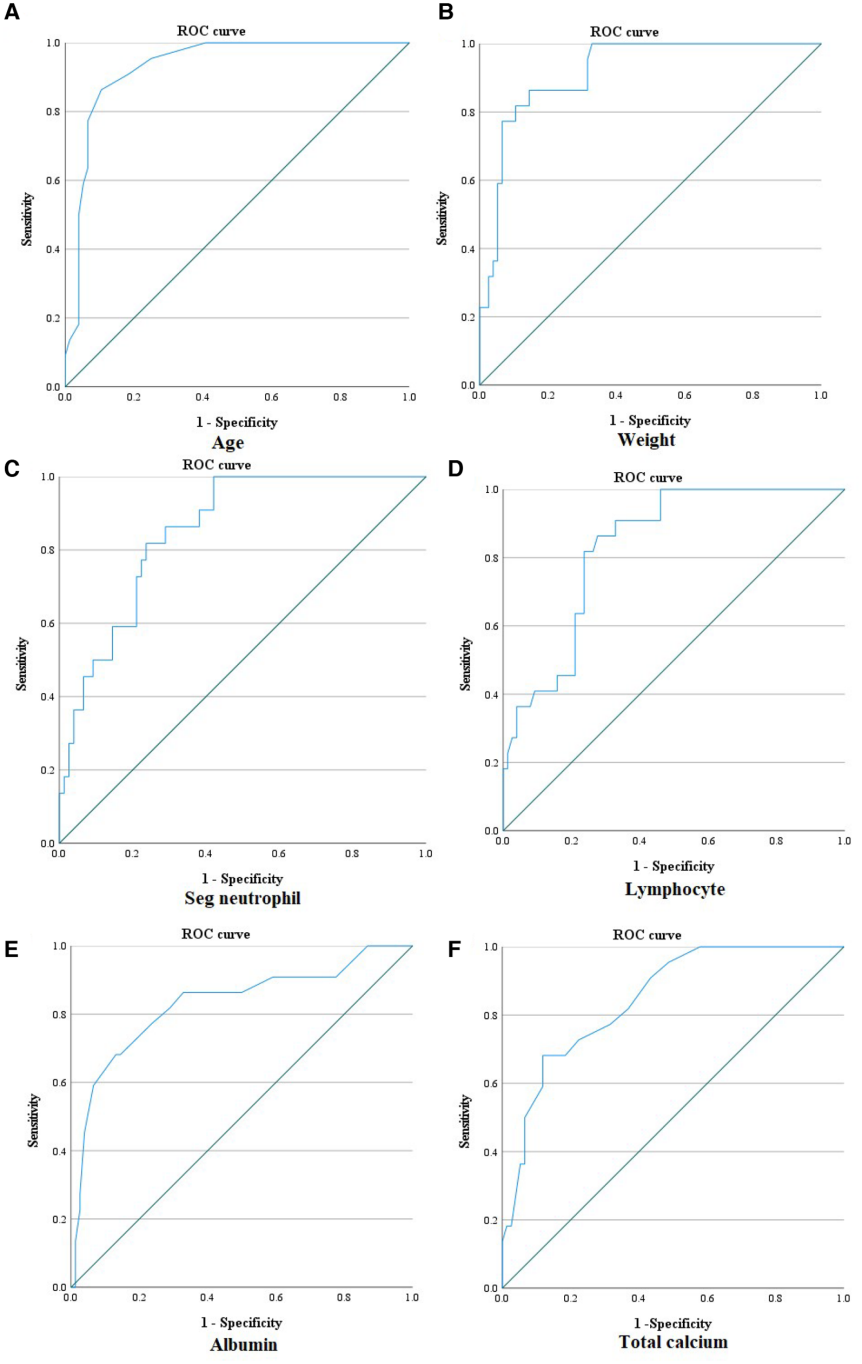
ROC curve of age, weight, segmented neutrophil percentage, lymphocyte percentage, albumin, and total calcium for diagnosing MIS-C. (**A**) ROC curve of age for diagnosing MIS-C [AUC: 0.931 (0.880–0.982); ≥4.5 years old: sensitivity 86.4%, specificity 89.5%]. (**B**) ROC curve of weight for diagnosing MIS-C [AUC: 0.917 (0.860–0.974); ≥16.75 kg: sensitivity 86.4%, specificity 85.5%]. (**C**) ROC curve of segmented neutrophil percentage for diagnosing MIS-C [AUC: 0.852 (0.775–0.929); ≥70.1%: sensitivity 81.8%, specificity: 76.3%]. (**D**) ROC curve of lymphocyte percentage for diagnosing MIS-C [AUC: 0.833 (0.752–0.915); ≤21.8%: sensitivity 81.8%, specificity 76.3%]. (**E**) ROC curve of albumin for diagnosing MIS-C [AUC: 0.830 (0.721–0.939); ≤3.85 g/dl: sensitivity 77.3%, specificity 76.3%]. (**F**) ROC curve of total calcium for diagnosing MIS-C [AUC: 0.846 (0.762–0.930); ≤9.25 mg/dl: sensitivity 72.7%, specificity 77.6%]. ROC curve, receiver operating characteristic curve; AUC, area under the curve.

Among the parameters that significantly differed between the KD and MIS-C groups, age had the largest AUC (0.931) in MIS-C diagnosis (0.880–0.982). Furthermore, the AUC value of height was 0.925 (0.880–0.982), weight was 0.917 (0.860–0.974), segmented neutrophils percentage was 0.852 (0.775–0.929), lymphocytes percentage was 0.833 (0.752–0.915), total calcium had 0.846 (0.762–0.930), and albumin was 0.830 (0.721–0.939), which were >0.800. Moreover, the sensitivity and specificity of the cut-off value exceeded 70.0% (Figure 2). With the age cut-off of ≥4.5 years, sensitivity and specificity were 86.4% and 89.5%, respectively. When the cut-off of the height was ≥111.55 cm, sensitivity and specificity were 81.8% and 90.8%, respectively. With the weight cut-off of ≥16.75 kg, sensitivity and specificity were 86.4% and 85.5%, respectively. When the cut-off of segmented neutrophils percentage was ≥70.1%, sensitivity and specificity were 81.8% and 76.3%, respectively. With a percentage of lymphocytes cut-off of ≤21.8%, the sensitivity and specificity of diagnosing MIS-C were 81.8% and 76.3%, respectively. When the cut-off of total calcium was ≥9.25 mg/dl, sensitivity and specificity were 72.7% and 77.6%, respectively. Albumin was ≤3.85 g/dl; therefore, sensitivity and specificity were 77.3% and 76.3%, respectively.

All parameters mentioned above, including age, height, weight, segmented neutrophils percentage, lymphocytes percentage, albumin, and total calcium, were included as independent variables for predicting MIS-C in the univariate and multivariate logistic regression. The results are presented in Table 3. All parameters significantly correlated with MIS-C in the univariate analysis. In multivariate analysis, albumin ≤3.85 g/dl (OR 56.55, 95% CI 3.64–878.96, *P = *0.004) was the only predictor of MIS-C. Additionally, we calculated the sensitivity and specificity of the combined tool of age and albumin. For age ≥4.5 years and albumin ≤3.85 mg/dl, sensitivity was 59.09%, and specificity was 96.05%. On the contrary, for age ≥4.5 years or albumin ≤3.85 mg/dl, sensitivity was 96.0%, and specificity was 71.23%.

**Table 3:** Results of logistic regression analysis for predicting MIS-C.

	Univariate analysis		Multivariate analysis	
	Odds ratio (95% CI)	*P*-value	Odds ratio (95% CI)	*P*-value
Age ≥4.5 years	53.83 (13.00–222.95)	<0.001	17.36 (0.68–443.49)	0.084
Height ≥111.55 cm	44.36 (11.69–168.31)	<0.001	6.07 (0.25–145.62)	0.266
Weight ≥16.75 kg	37.42 (9.46–148.04)	<0.001	0.943 (0.03–28.07)	0.971
Seg neutrophil ≥70.1%	14.50 (4.34–48.41)	<0.001	3.80 (0.32–44.78)	0.290
Lymphocyte ≤21.8%	14.50 (4.34–48.41)	<0.001	1.86 (0.14–25.51)	0.644
Albumin ≤3.85 g/dl	11.80 (3.80–36.67)	<0.001	29.52 (3.15–276.55)	**0**.**003**
Total calcium ≤9.25 mg/dl	8.04 (2.80–23.03)	<0.001	0.24 (0.02–2.51)	0.234

Seg neutrophil, segmented neutrophil.

Additionally, we performed an unpaired *t* test and multiple variable analysis after propensity score matching with age, height, and weight for even sample distribution and to estimate the parameters that most effectively predict MIS-C in children whose age can imply a diagnosis of both KD and MIS-C. The results are presented in Tables 4–6. Even after propensity score matching with age, height, and weight, the mean values were higher in group 1. The segmented neutrophils percentage, lymphocytes percentage, and albumin levels were significantly different after propensity score matching. Finally, only albumin could predict MIS-C based on the results of multiple logistic regression after propensity score matching with age, height, and weight.

**Table 4:** Comparison of the mean values of independent variables after propensity score matching with age, height, and weight.

	Group 1 (*n* = 22)	Group 2 (*n* = 22)	*P*-value
Age (years)	8.5 ± 3.8	5.0 ± 3.8	**0** **.** **004**
Height (cm)	134.9 ± 25.2	112.1 ± 21.6	**0**.**002**
Weight (kg)	35.0 ± 17.5	21.8 ± 12.0	**0**.**006**
BMI percentile	53.3 ± 36.5	59.2 ± 30.9	0.575
Seg neutrophils (%)	76.6 ± 9.5	61.5 ± 19.3	**0**.**003**
Lymphocytes (%)	16.0 ± 8.2	26.4 ± 17.0	**0**.**015**
Albumin (g/dl)	3.5 ± 0.9	4.1 ± 0.4	**0**.**004**
Calcium (mg/dl)	8.5 ± 1.8	9.3 ± 0.5	0.050

Seg neutrophil, segmented neutrophil.

**Table 5:** Multiple logistic regression results of independent variables after propensity score matching with age, height, and weight.

	Odds ratio (95% CI)	*P* value
Seg neutrophil (%)	1.08 (0.92–1.30)	0.310
Lymphocyte (%)	1.00 (0.82–1.25)	0.896
Albumin (mg/dl)	0.20 (0.03–0.69)	0.017

Seg neutrophil; segmented neutrophil.

**Table 6:** Treatment approaches and outcomes in patients with MIS-C.

Treatment approaches	
None	22.7% (5/22)
IVIG only	41.7% (5/12)
IVIG + methylprednisolone	52.9% (9/17)
Concomitant use	55.6% (5/9)
mPD pulse d/t treatment failure	44.4% (4/9)
Treatment failure on the first IVIG^a^	23.5% (4/17)
Use of inotrope	13.6% (3/22)
Aspirin	100.0% (22/22)
**Outcome**	
Discharge from hospital	100%
Death	0

^a^ Treatment failure is defined as persistent fever for 36 h after IVIG.

### Treatment approaches and outcomes in patients with MIS-C

Table 6 presents treatment approaches to MIS-C and treatment outcomes. In 22.7% of patients with MIS-C, fever subsided without treatment. All patients were administered aspirin to prevent thromboembolic events regarding the less need for IVIG (intravenous immunoglobulin) treatment. Inotrope was required in 13.6% of patients to maintain optimal blood pressure. Additionally, no patient required mechanical ventilation. Among the IVIG-treated group, steroid treatment was combined in 52.9% of patients, and 55.6% of patients treated with IVIG and steroids used methylprednisolone concomitantly due to hemodynamic instability. Treatment failure occurred in 23.5% of patients who received the first treatment with IVIG. In total, refractory cases occurred in 18.2% of the patients, and there was no significant difference in the refractory case incidence of the first KD treatment with IVIG (19.7%, *P *= 1.000). On the contrary, the rate of corticosteroid use was higher in group 1 than in group 2 (40.9 vs. 15.8%, *P *= 0.018). No death occurred in our study.

### Echocardiographic changes 1 month after diagnosis of MIS-C

**Figure 3:**
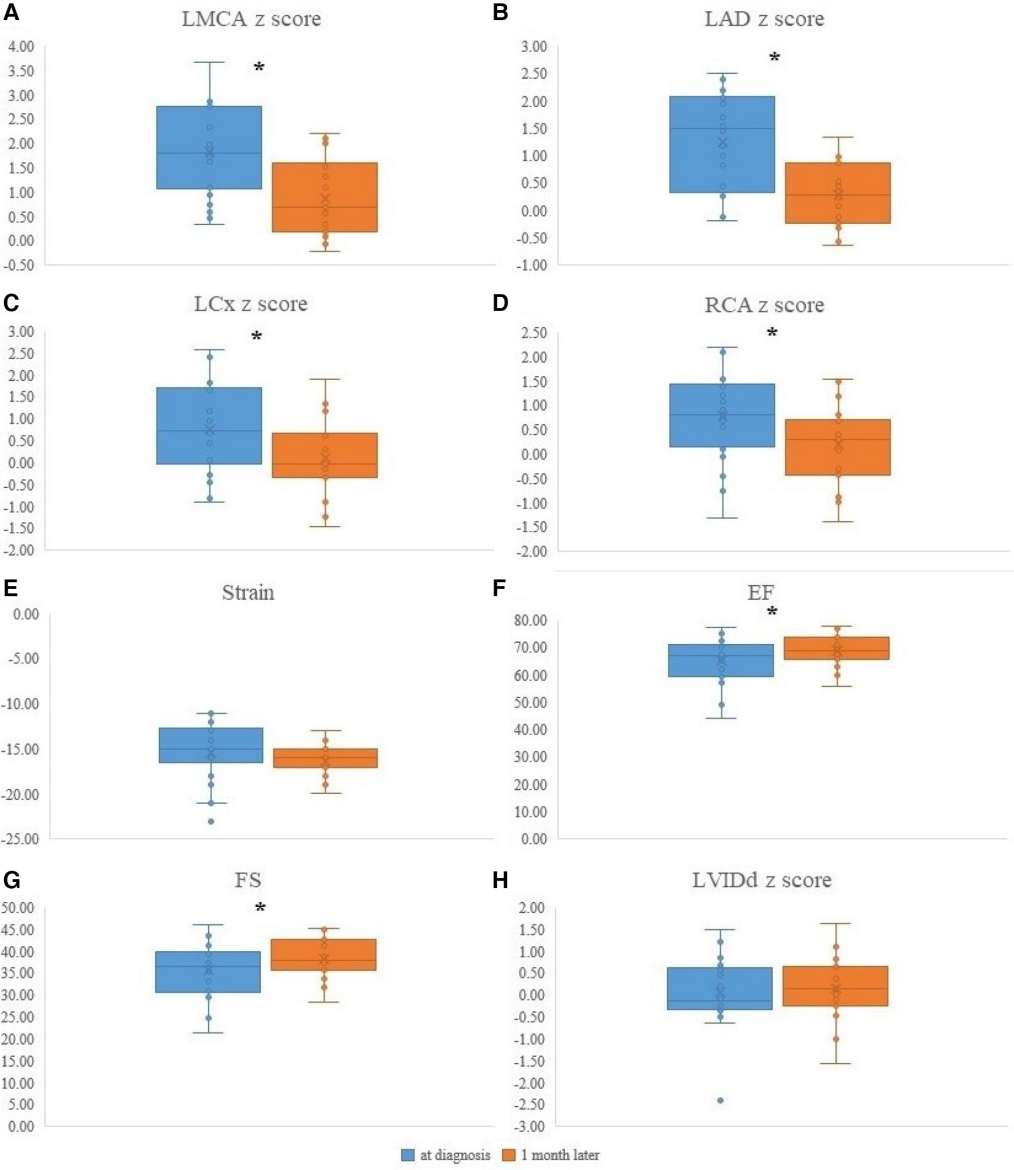
The echocardiographic data of MIS-C patients at diagnosis and 1 month after MIS-C diagnosis. (**A**) LMCA *z*-score changes at diagnosis and 1 month after diagnosis (1.8 ± 1.0 to 0.9 ± 0.8, *P < *0.001). (**B**) LAD *z*-score changes at diagnosis and 1 month after diagnosis (1.2 ± 0.9 to 0.3 ± 0.6, *P *< 0.001). (**C**) LCx *z*-score changes at diagnosis and 1 month after diagnosis (0.8 ± 1.1 to 0.1 ± 0.9, *P *= 0.008). (**D**) RCA *z*-score changes at diagnosis and 1 month after diagnosis (0.8 ± 0.8 to 0.2 ± 0.8, *P *= 0.017). (**E**) Apical 4-chamber LV longitudinal strain changes at diagnosis and 1 month after diagnosis (−15.6 ± 3.5 to −16.4 ± 1.7, %, *P *= 0.359). (**F**) EF changes at diagnosis and 1 month after diagnosis (64.8 ± 8.6 to 68.9 ± 6.1, %, *P *= 0.035). (**G**) LV FS changes at diagnosis and 1 month after diagnosis (35.4 ± 6.4 to 38.4 ± 4.7, %, *P *= 0.046). (**H**) LVIDd *z*-score changes at diagnosis and 1 month after diagnosis (0.2 ± 0.6 to 0.2 ± 0.8, *P *= 0.889). LMC, Left main coronary artery; LAD, Left anterior descending artery; LCx, Left circumflex artery; RCA, Right coronary artery; LV, Left ventricle; A4C, Apical 4 chamber; EF, Ejection fraction; FS, Fractional shortening; LVIDd, diastolic left ventricular internal diameter. *P < 0.05.

Echocardiographic changes 1 month after MIS-C diagnosis are illustrated in Figure 3. All coronary arteries *z*-scores were decreased 1 month after MIS-C diagnosis. The decrease in *z*-scores of each coronary artery was as follows: LMCA (1.8 ± 1.0 to 0.9 ± 0.8, *P < *0.001), LAD (1.2 ± 0.9 to 0.3 ± 0.6, *P *< 0.001), LCx (0.8 ± 1.1 to 0.1 ± 0.9, *P *= 0.008), and RCA (0.8 ± 0.8 to 0.2 ± 0.8, *P *= 0.017). EF (64.8 ± 8.6 to 68.9 ± 6.1, *P *= 0.035) and LV FS (35.4 ± 6.4 to 38.4 ± 4.7, *P *= 0.046) improved 1 month after MIS-C diagnosis. No change was observed in the apical 4-chamber LV longitudinal strain and LVIDd *z*-scores between the two time points.

### Reproducibility testing

One investigator (J.Y.) performed the echocardiography. No interobserver coefficient test was done. The intraobserver coefficient in apical 4-chamber longitudinal strain was 0.946 (0.865–0.974) in this study.

## Discussion

This is the first study to differentiate MIS-C from KD in Korea. We analyzed the clinical difference between MIS-C and KD groups, especially in the area with a high prevalence of KD. Additionally, we observed the cardiac function change over time. As previously reported (5, 16, 17), MIS-C patients were older than KD patients. Even though overweight was found in about a quarter of the MIS-C group in a previous study (18), our study showed no significant difference in BMI percentile between the two groups. Additionally, we showed the clinical characteristic difference of KD features between the MIS-C and KD groups. Rash and extremity change were infrequently observed in the MIS-C group. Segmented neutrophils percentage, CRP, and PT (seconds) were elevated in the MIS-C group, while lymphocytes percentage, albumin, potassium, chloride, phosphorus, and total calcium levels were lower in the MIS-C group compared with those in the KD group. In the echocardiographic examination, the absolute values of LV strain, EF, and FS were lower in the MIS-C group. After compounding the age factor, albumin was the only predictor of MIS-C. All coronary arteries *z*-scores were decreased 1 month after MIS-C diagnosis. Additionally, EF and FS increased 1 month after MIS-C diagnosis.

The prevalence of MIS-C is relatively low in Asians (4); however, with the Omicron outbreak in January 2022, COVID-19 infections surged (19). Compared to previous events, COVID-19 is becoming endemic. As SARS-CoV-2 infections increase and the number of children with COVID-19 history increases, MIS-C prevalence is expected to increase, like the New York study (3). MIS-C occurs in <1% of pediatric COVID-19 patients. However, since approximately 80% of MIS-C occurs in the cardiovascular system, MIS-C will likely become a major acquired heart disease in children following KD (3, 4).

In Korea, KD prevalence is the second highest in the world (20). KD is known to occur mainly in children before the age of 5; nonetheless, it is common for children to develop it after the age of 5 (21). In contrast, MIS-C often occurs after the age of 5; however, children aged 2–3 years were included in this study (22). It is challenging to diagnose MIS-C, differentiating it from KD in a 2–3-year-old child, especially with COVID-19 history. For diagnosing MIS-C, cardiac markers, coagulation tests, or serologic tests for SARS-CoV-2 are essential to determine whether other organs have been involved. However, it is impossible to run several blood tests on children who are not adolescents, and there will be controversy regarding its effectiveness. Therefore, this paper suggested a direction regarding the utility of additional laboratory tests when albumin is ≤3.85 g/dl at the time of initial diagnosis, even for 2–3 years old children.

From the aspect of more frequently observed electrolyte imbalance in MIS-C than in KD, first, total calcium level goes with albumin level (23). The total calcium level was not a significant factor after multiple regression analysis when analyzed with the albumin level. Potassium, phosphorus, and chloride levels were also decreased in the MIS-C group. Among these, the phosphorus level can vary according to the age of pediatric patients (24); however, along with hypochloremia, hypokalemia, and hypophosphatemia, this can be caused by gastrointestinal loss, such as vomiting and diarrhea with dehydration (25). It can be explained by the premise that gastrointestinal symptoms are prominent in the MIS-C group (5, 16, 17).

There are several reports of mid-term follow-up data on giant coronary aneurysms in MIS-C (26). The resolution of left ventricular ejection fraction and coronary artery aneurysms are reported a month after diagnosis; nonetheless, diastolic dysfunction persisted during the subacute mean period of 5 days (27, 28). In our study, EF and FS were improved one month after diagnosis, although apical 4 chamber LV strain did not improve. Furthermore, a reduction in the *z*-scores of the coronary arteries was observed. We observed this trend even in patients without coronary artery dilatation. This could be due to the ethnic background that early detection of MIS-C in Korea was possible owing to the high prevalence of KD. As a result, there is a possibility that we included fewer patients who had elevated troponin-T and required inotropes than in previous studies. However, prolonged follow-up or additional patient enrolment is required to verify this theory.

IVIG alone is recommended as the primary KD treatment in the current guideline; however, there is a debate concerning MIS-C (29). In our patients, no significant difference was observed in the refractory case rate between the MIS-C and KD groups; however, in the MIS-C group, 2 mg/kg methylprednisolone was administered to 55.6% of patients as the initial treatment; hence, it should be cautiously interpreted. Also, MIS-C mainly causes fatal diseases, such as hemodynamic instability or death (27). However, the rate of inotrope or ventilator use in our study was significantly lower than in previously published studies (27), which may be characteristics of MIS-C occurring in Asia or MIS-C-suspected cases before it progresses to areas with high KD prevalence. Patients can be diagnosed early when they promptly present to a tertiary hospital. Indeed, there was no significant difference in the duration of fever before treatment in the KD and MIS-C groups.

Limitations of this study were that it was a retrospective, single-center study, and the sample size was small. Furthermore, although the diagnosis was made according to the criteria, there may be a debate about the final diagnosis. Therefore, a nationwide multicenter study is needed in the future. Nevertheless, this study is significant because it revealed the diagnostic points in differentiating KD from MIS-C in a high-KD prevalence area.

In conclusion, KD and MIS-C were most easily distinguished using albumin values. On echocardiography, significant differences in apical 4-chamber LV longitudinal strain, EF, and FS were observed between the MIS-C and KD groups. A change in the size of coronary arteries, EF, and FS was identified 1 month after diagnosis.

## Data Availability

The original contributions presented in the study are included in the article/Supplementary Materials, further inquiries can be directed to the corresponding author/s.
